# Organ-on-chip (OoC) and nano-biomaterials: next generation of precision oral and dental healthcare research

**DOI:** 10.1186/s12951-026-04410-5

**Published:** 2026-04-25

**Authors:** Al-Hassan Soliman Wadan, Dana Saeed Abd-Elmonem El-Gemaie, Asser Magdi Bayoumi, Mohamed Mahfouz Zein, Mohamed Abd El Sattar Ahmed, Ahmed M. El-Khawaga, Mohamed M. Nagy

**Affiliations:** 1 Department of Oral Biology, Faculty of Dentistry, Galala University, Galala Plateau, Attaka, Suez, Egypt; 2 Department of Endodontics, Faculty of Dentistry, Galala University, Galala Plateau, Attaka, Suez, Egypt; 3 Department of Oral and Maxillofacial Surgery, Faculty of Dentistry, Galala University, Galala Plateau, Attaka, Suez, 15888 Egypt; 4 Department of Fixed Prosthodontics, Faculty of Dentistry, Galala University, Galala Plateau, Attaka, Suez, 15888 Egypt; 5 Department of Periodontology, Faculty of Dentistry, King Salman International University, South Sinai, El-Tor, Egypt; 6 Department of Basic Medical Sciences, Faculty of Medicine, Galala University, Galala City, Suez, 43511 Egypt; 7 Department of Endodontics, Faculty of Dentistry, Ain Shams University, Cairo, Egypt

**Keywords:** Organ-on-chip (OoC), Oral-on-chip, Biofilm, Microfluidics, Multi-tissue co-culture, Sustainable healthcare, Precision medicine, Microphysiological systems

## Abstract

Periodontitis and dental caries are the most widespread non-communicable oral disorders worldwide, affecting approximately 3.5 billion people worldwide. Nevertheless, existing in vitro models do not accurately emulate the intricate interactions between the oral microbiome, the host immune response, and the fluctuating physicochemical environment. This lack of physiologically realistic models makes it extremely difficult to understand disease mechanisms and to design effective therapies. While organ-on-a-chip technology has revolutionized biomedical research, its application in oral healthcare and dental precision medicine remains fragmented, often lacking the integration of functional materials necessary for precise monitoring and treatment. Herein, this review offers a comprehensive overview of the oral microenvironment, emphasizing the essential biophysical signals of the tooth surface, gingival crevice, and periodontal contact that must be replicated. We rigorously assess current oral-on-a-chip systems, pinpointing their deficiencies in replicating multi-tissue interactions and real-time monitoring. New oral-on-chip and organ-on-chip systems need to be made as a next-generation platform that combines microfluidics with advanced/ functional nanomaterials that bridge this gap. This suggested system incorporates sustainable, biodegradable, and bio- and nanomaterials with integrated nanosensors for real-time monitoring of pH and cytokines, providing a comprehensive solution for modeling oral illnesses. Our primary objective is to establish a framework for creating high-fidelity, sustained in vitro oral models that can expedite the advancement of precision oral healthcare.

**Graphical abstract:**

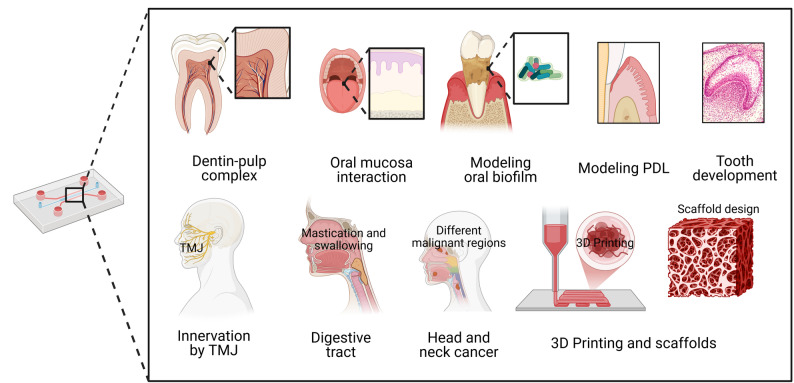

## Introduction

Oral diseases, particularly dental caries and periodontitis, pose a massive global health burden, affecting nearly half of the world’s population according to the World Health Organization (WHO) [1]. Untreated dental caries in permanent teeth is the most common health condition globally, while severe periodontal disease, which may result in tooth loss, affects approximately 19% of the global adult population [2]. Beyond the immediate impact on oral function and quality of life, these conditions are increasingly linked to systemic diseases such as cardiovascular disease, diabetes, and adverse pregnancy outcomes [3]. Additionally, oral cancer remains a significant cause of mortality worldwide, with 5-year survival rates remaining stagnant despite therapeutic advances [4]. The complexity of the oral cavity, characterized by a unique interface between hard and soft tissues, a diverse microbiome [5–7], and constant environmental challenges, necessitate the use of advanced research tools and integration of nanomedicines to unravel disease mechanisms and develop targeted therapies [8].

Traditional in vitro models, such as 2D monolayer cultures and static biofilm assays, fail to capture the dynamic, multicellular nature of the oral microenvironment, especially the complex array of cell types found within the dental pulp alone [9]. They lack critical physiological features such as fluid shear stress from saliva, pH fluctuations, and the structural complexity of the host-microbiome interface [10]. Animal models, while offering systemic context, are limited by interspecies differences in oral anatomy, microbiome composition, and immune responses, raising concerns about their translational value and ethical implications [11, 12]. Consequently, there is a critical need for advanced in vitro platforms that accurately mimic human oral physiology and pathology, facilitating high-throughput drug screening and personalized medicine.

**Fig. 1:**
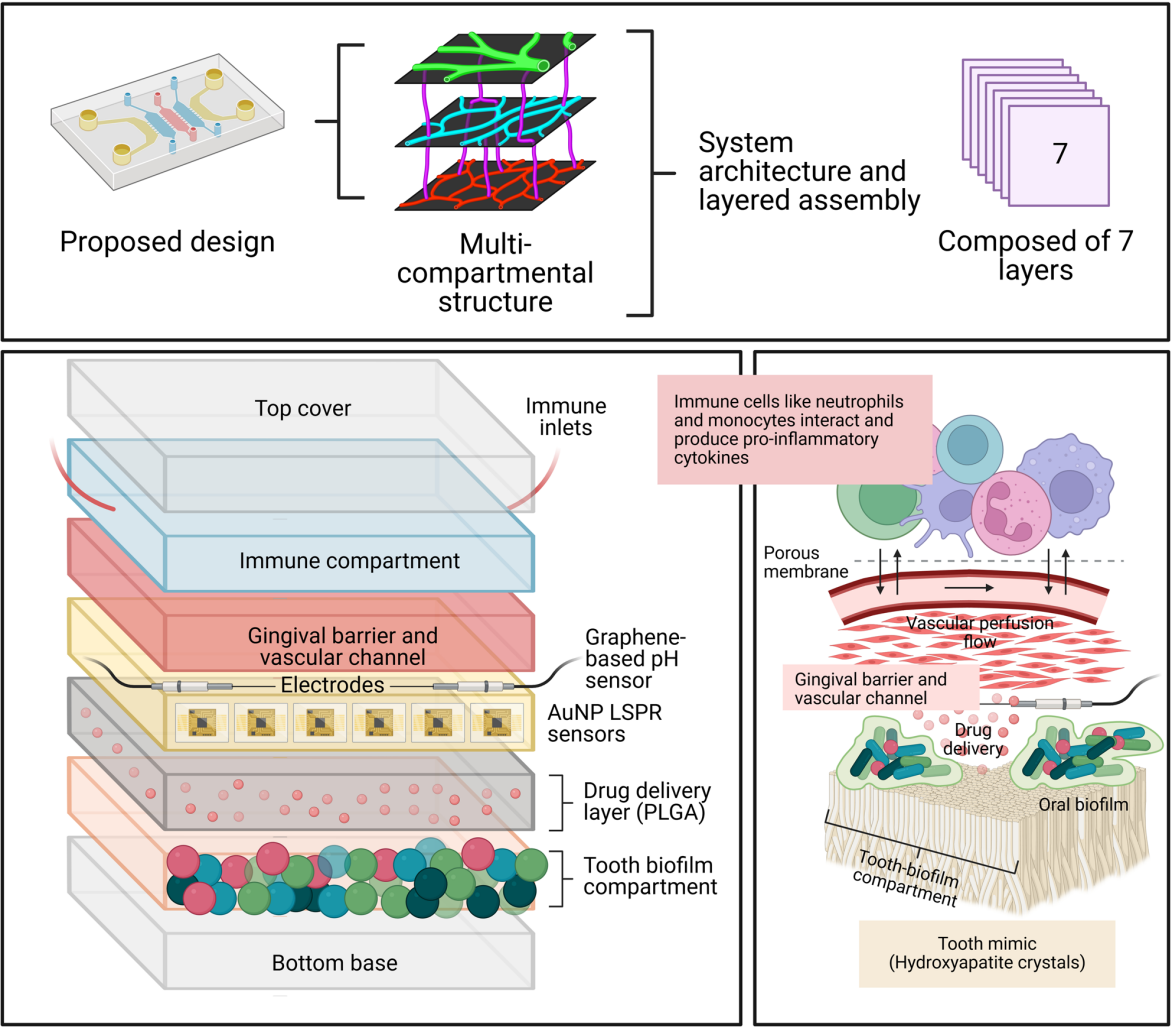
Integrated smart oral-on-a-chip platform for modeling biofilm-immune-vascular interactions. (Top) Conceptual overview of the proposed multi-compartmental design, composed of a stacked architecture containing seven functional layers. (Bottom Left) A detailed vertical schematic of the fully stacked device assembly, clearly labeling each of the seven layers from top cover to bottom base, which includes the immune compartment with specialized immune cell inlets, the gingival barrier and vascular channel (integrated with electrodes and AuNP sensors for electronic and optical monitoring), a dedicated drug delivery layer, and the tooth biofilm compartment. (Bottom Right) An illustrative cross-section showing the dynamic interactions within the system: immune cell filtration and cytokine production across a porous membrane in the upper compartment; vascular perfusion; and the targeted delivery of a drug to an oral biofilm cultivated on a hydroxyapatite tooth-mimic surface at the bottom. All figures have been created using BioRender.com

Organ-on-a-chip (OoC) technology, and most recently Human-on-a-chip [13], also known as microphysiological systems (MPS), has emerged as a transformative approach to bridge the gap between traditional in vitro models and clinical reality [14–16]. By leveraging microfluidics and tissue engineering [17], OoC platforms can recapitulate organ-level functions (see Fig. 1), such as the brain [18–20], Parkinson’s disease (PD) pathogenesis and treatment approaches [21], the blood-brain-barrier (BBB) [22], the cornea [23], the liver and liver spheroids [24, 25], the pancreas [26], pancreas-liver combination [27], heart [28], cardiac endothelial tumor [29], cardiac pump [30], cardiac spheroids [31], lung [32], lung alveolus [33], lung cancer [34], radiation-induced lung injury [33], combination of lung-liver interaction [35, 36], elucidation of the pathophysiology of COVID-19 using lung-on-chip model [37], lung endothelial dysfunction [38], joint-on-chip [16], cell-cell interactions, and dynamic mechanical cues. While significant progress has been made in developing brain-on-chip [39], lung-on-chip [40], liver-on-chip [41], and gut-on-chip [42], the development of comprehensive oral-on-a-chip systems has lagged. Current oral chips predominantly focus on single aspects, such as biofilm formation or gingival epithelial barrier function [43, 44], often neglecting the complex crosstalk between the tooth, periodontium, and immune system. Furthermore, the potential of integrating functional nanobiomaterials for high-sensitivity sensing and targeted delivery remains largely unexplored in this domain. By proposing this design in Fig. 1, nanosensors are strategically integrated into the microfluidic channels and porous membranes that interface directly with distinct tissue-specific layers, such as those that house iPSC-derived ameloblast organoids. AuNPs are deposited onto these microelectrode arrays to significantly amplify signal conductivity, thereby enhancing the electrochemical detection of cellular metabolites, localized pH fluctuations, and specific biomarkers secreted by differentiating cells. This seamless integration of biological scaffolds with AuNP-enhanced sensing layers enables highly sensitive, real-time, and non-destructive monitoring of the physiological microenvironment during enamel regeneration.

This review aims to address these gaps by proposing a convergence of OoC technology for precision oral healthcare. We first describe the complex microenvironmental features of key oral tissues, including the tooth surface, gingival crevice, and periodontal ligament. Subsequently, we provide a critical analysis of existing oral-on-chip platforms, highlighting their achievements and fundamental limitations. Central to our thesis is the integration of nanomaterials to reveal smart functionalities, such as real-time sensing and stimuli-responsive drug release. Finally, we propose a conceptual design for a Smart “*Oral-on-Chip”* that incorporates sustainable materials and multi-tissue integration, offering a visionary roadmap for the future of oral disease modeling and drug discovery.

## The oral microenvironment

### The tooth surface microenvironment

The oral cavity represents a uniquely challenging physiological environment where mineralized structures penetrate a mucosal barrier, creating distinct micro-niches inhabited by a complex microbiome [45]. Constructing a valid OoC requires recreating these specific tissue microenvironments. The tooth surface presents the primary interface for microbial colonization. Enamel, the hardest substance in the human body, consists of 96% mineral, primarily hydroxyapatite crystals organized into a complex prismatic architecture [46]. This structure has an elastic modulus of approximately 80 GPa, which is essential for withstanding masticatory forces. Immediately upon exposure to the oral cavity, enamel is coated by the acquired pellicle, a proteinaceous film derived from saliva that dictates initial bacterial adhesion and biofilm formation [47]. Under cariogenic challenge, acidogenic bacteria within the biofilm metabolize fermentable carbohydrates, creating localized pH gradients that can drop below the critical pH of 5.5, leading to demineralization [45, 48]. Underlying the enamel is dentin, a tubular structure housing the odontoblastic processes that provide mechanosensory feedback, a feature often overlooked in current tooth microchip models [47].

### The gingival crevice microenvironment

The gingival crevice, or sulcus, is a narrow space between the tooth surface and the free gingiva, lined by the sulcular and junctional epithelia. The junctional epithelium forms a unique semipermeable seal at the tooth surface, characterized by a rapid turnover rate and wide intercellular spaces that facilitate the passage of gingival crevicular fluid (GCF) and immune cells [49]. The GCF flows at a rate of 0.5-2 µL/h, flushing the crevice and carrying cytokines (IL-1β, IL-6, TNF-α), enzymes (MMPs), and immune cells (PMNs, macrophages, dendritic cells) [50]. This fluid dynamic is crucial for maintaining homeostasis and shifts dramatically during inflammation. Recent research has been using nanomaterials to repurpose macrophage metabolism [46]. Mechanically, the gingival tissues are subjected to significant forces during mastication, ranging from 500 to 700 N in the molar region, which influences epithelial barrier integrity, fibroblast remodeling, and ECM stiffness [51]. ECM stiffness directly regulates immune homeostasis in gingival fibroblasts. Stiffer matrices, mirroring healthy tissue, promote an anti-inflammatory, tissue-maintaining phenotype by upregulating ECM synthesis genes. In contrast, softer matrices characteristic of periodontal disease induce pro-inflammatory responses and tissue degradation [52].

Using a tunable collagen-alginate hydrogel model, researchers confirmed that soft matrices exacerbate toll-like receptor-mediated inflammation in human GFs, a stiffness-dependent response directed by the non-canonical NFκB pathway and epigenetic nuclear organization [53].

### The periodontal ligament-bone interface

The periodontal ligament (PDL) is a specialized connective tissue situated between the tooth root and the alveolar bone. It is composed of highly organized collagen fiber bundles, known as Sharpey’s fibers, which are inserted into the cementum and bone. These fibres provide support and shock absorption. The PDL is highly vascularized and innervated, functioning as a sensory organ for occlusal detection. Alveolar bone remodeling is tightly regulated by the balance between osteoblasts and osteoclasts, a process sensitive to mechanical loading [54]. Orthodontic forces induce mechanotransduction pathways within PDL cells, triggering bone resorption on the compression side and formation on the tension side [49, 55–58]. Recreating this mechanobiological coupling is essential for modeling the progression of periodontitis and orthodontic tooth movement.

### The salivary microenvironment

Saliva is the omnipresent medium of the oral cavity, composed of water, electrolytes, mucins, antimicrobial peptides, and enzymes [59]. It provides lubrication, buffering capacity via bicarbonate and phosphate systems, and immune protection [60, 61]. The continuous flow of saliva generates fluid shear stress ranging from 0.01 to 1 dyn/cm² on oral surfaces, which regulates bacterial adhesion and epithelial phenotype [62, 63]. Furthermore, salivary flow exhibits circadian variations that influence the oral pH and clearance rates [64, 65].

## Current oral-on-chip systems

### Fabrication of organ-on-chip platforms

Organ-on-a-chip systems are advanced in vitro microphysiological platforms designed to mimic the complex 3D microenvironments, dynamic mechanical forces, and functional tissue-tissue interfaces of human organs, operating fundamentally on the principles of microfluidics to manipulate microliter to picoliter fluid volumes [66]. The fabrication of these complex devices has historically relied on photolithography to pattern precise microstructures on substrates using photoresists like SU-8, which subsequently serve as master molds for replication techniques such as soft lithography. Soft lithography typically utilizes PDMS due to its high optical transparency, gas permeability, and capability to replicate sub-micron features, allowing the creation of flexible porous membranes and compartmentalized microchannels [67]. To overcome inherent PDMS-related limitations such as the unwanted absorption of small hydrophobic drug molecules, recent fabrication advancements have introduced hot embossing and microinjection molding to shape resilient thermoplastics (e.g., polycarbonate and cyclic olefin copolymers), offering superior scalability, reduced small-molecule absorption, and industrial mass production capabilities [68].

Furthermore, emerging barrier-free engineering techniques, including hydrogel photopatterning, phaseguide design, and 3D bioprinting, aim to eliminate inert artificial membranes entirely, thereby promoting direct cellular crosstalk, native physiological gradients, and enhanced biomimicry [69]. When comparing performance, OOCs drastically outperform conventional static 2D cell cultures, which fail to replicate in vivo cellular architectures and fluid dynamics, as well as 3D spheroids that lack dynamic multiscale interfaces and vascular perfusion. Crucially, OOCs also offer profound advantages over traditional animal models by avoiding interspecific genetic and physiological discrepancies that often lead to inaccurate predictions of human drug pharmacokinetics and pharmacodynamics, simultaneously alleviating ethical concerns and reducing the exorbitant costs of prolonged clinical trials [70].

In practice, OOC technology has been deployed across numerous detailed application cases. For instance, the landmark breathing lung-on-a-chip utilizes a compartmentalized micro-porous membrane subjected to cyclic, vacuum-driven stretching to simulate physiological respiratory motions, enabling precise evaluations of pulmonary nanotoxicity and viral infections like SARS-CoV-2 [71]. Similarly, liver-on-a-chip platforms, integrating iPSC-derived hepatocytes, Kupffer cells, and endothelial cells under continuous perfusion, are routinely utilized to predict human-specific drug-induced liver injury with high sensitivity, while BBB-on-a-chip models facilitate the meticulous analysis of CNS drug penetrability [27]. Finally, the evolution of single-organ models has culminated in highly sophisticated multi-organ-on-a-chip systems, which fluidically interconnect distinct organ modules to simulate complex systemic interactions, comprehensive oral drug absorption, metabolism, off-target toxicity, and metastasis, collectively paving the way for highly predictive preclinical screening and personalized medicine [72].

### Single-layer biofilm chips

**Fig. 2:**
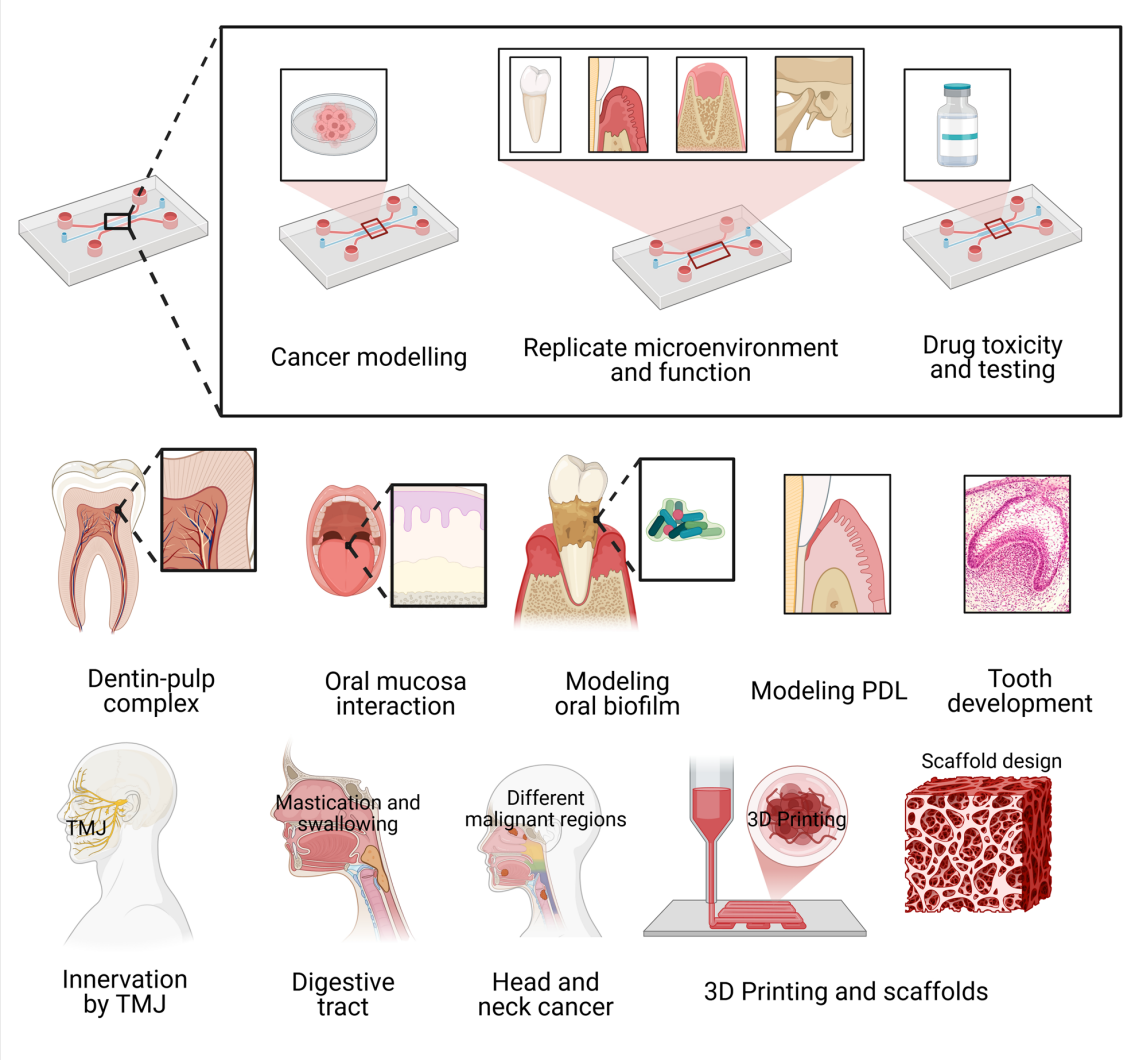
Organ-on-a-chip devices have been used to study cancers by modeling cancer tissues in these microphysiological systems, by replicating the tumor microenvironment, and by drug toxicity and drug testing. Additionally, these devices have been used to model the dentin-pulp complex [85–89] and oral mucosa interactions with metals [90], dental materials and bacteria [91], and silver diamine fluoride [89], saliva, and oral biofilms [92–96], PDL modeling [97], stages of tooth development, modeling cells like the SCAFs [85, 98], TMJ-tooth innervation [99], digestion and mastication [100], various head and neck cancers [101, 102], and the scaffolding of many organs. All figures have been created using BioRender.com

Recent years have seen the emergence of microfluidic devices designed to simulate various tissues and organs (see Fig. 2) [14, 73], as well as specific aspects of the oral environment, including biofilm, soft tissue, and hard tissue models [64, 74–78] (see Table 1). Microfluidic flow cells have been widely used to study oral biofilm formation and antimicrobial responses. Several studies have developed chips with parallel channels to culture *Staphylococcus aureus*,* Streptococcus mutans*, or multi-species microcosms under controlled flow conditions [79, 80]. These platforms enable high-resolution imaging of biofilm architecture and quantification of metabolic activity. While these systems offer superior control over fluid dynamics compared to static well plates, they typically utilize glass or plastic substrates that fail to mimic the physicochemical properties of enamel or dentin, and they lack the crucial interface with host cells.

Recent advances in dental organ-on-chip technologies have enabled increasingly sophisticated in vitro models that recapitulate key structural and biological features of the dentine-pulp-implant interface [76, 81]. The tooth-on-chip platform developed by Cordiale et al. integrates dentine-derived materials, odontoblast-like cells, DPSCs, endothelial cells, and trigeminal neurons within a multi-compartment microfluidic architecture, successfully reproducing vascularized and innervated pulp-like tissue organization [82]. Similarly, microfluidic innervation models by Pagella and colleagues demonstrated the dynamic interaction between the trigeminal ganglia and developing tooth tissues, providing mechanistic insights into tooth innervation [82, 83]. Complementary approaches, such as dentin-on-a-chip systems based on GelMA hydrogels and apical papilla stem cells, further enabled controlled studies of odontogenic differentiation and dentine-cell interactions under defined microenvironmental conditions [81, 84].

Despite these advances, several methodological and biological limitations remain. Most platforms rely on simplified cellular compositions, frequently employing HUVECs as generic endothelial models and lacking immune cells or tissue-specific vascular phenotypes, which may limit physiological relevance [103, 104], particularly for inflammatory or infectious conditions [82]. Material-related constraints also persist. For instance, the Polydimethylsiloxane (PDMS)- based devices are prone to adsorption of small molecules, potentially altering effective drug concentrations and complicating quantitative therapeutic testing, as highlighted in angiogenesis- [105], in the dental pulp [106], and implant-on-chip studies [107–109]. Additionally, reliance on dentine particles or extracted dental tissues introduces batch-to-batch variability and reduces standardization, while complex fabrication processes may limit scalability and reproducibility across laboratories. Computational diffusion models [107–109], although informative, do not always fully match experimental transport behavior, underscoring ongoing challenges in accurately mimicking in vivo mass transport dynamics.

**Fig. 3:**
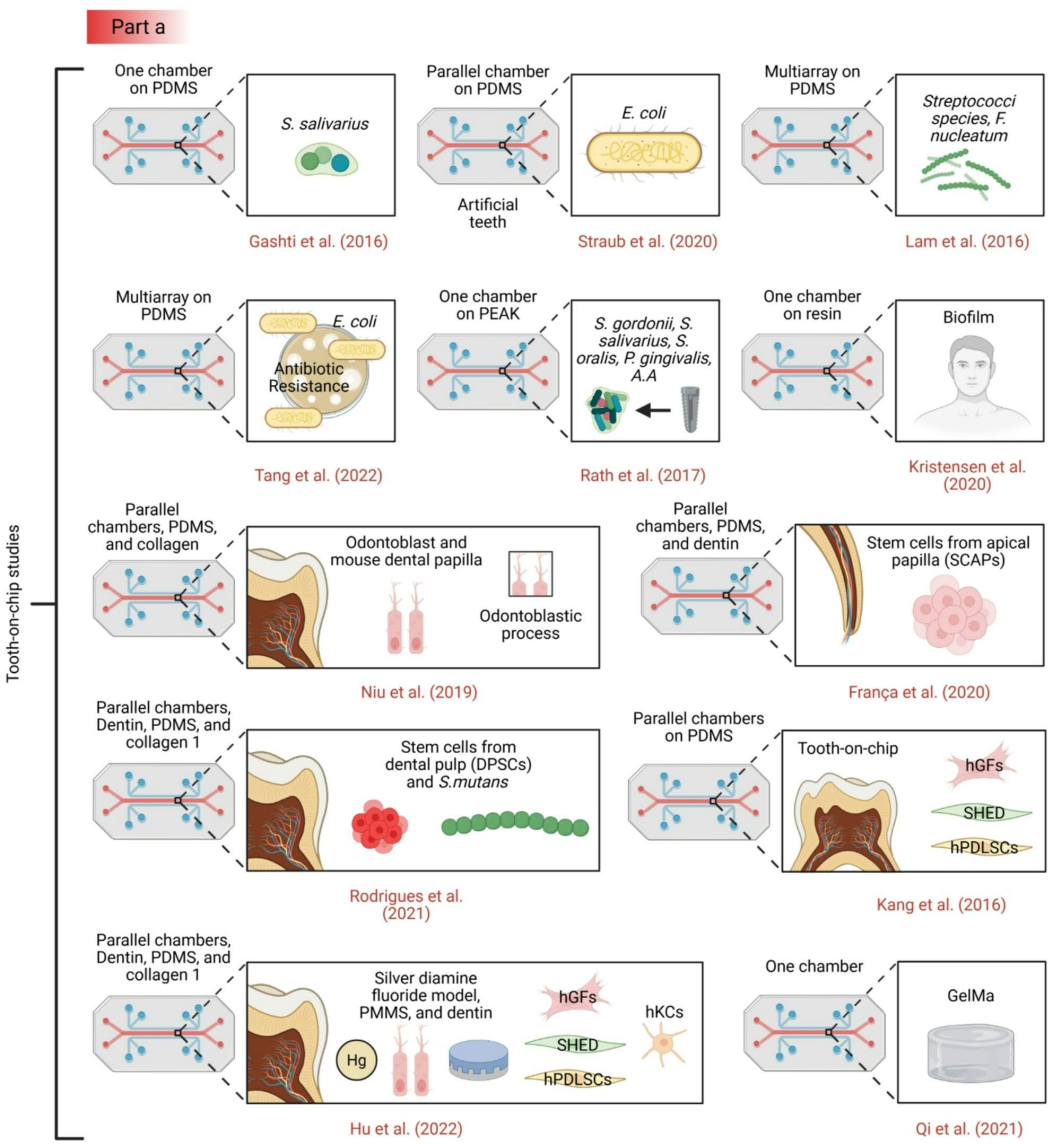

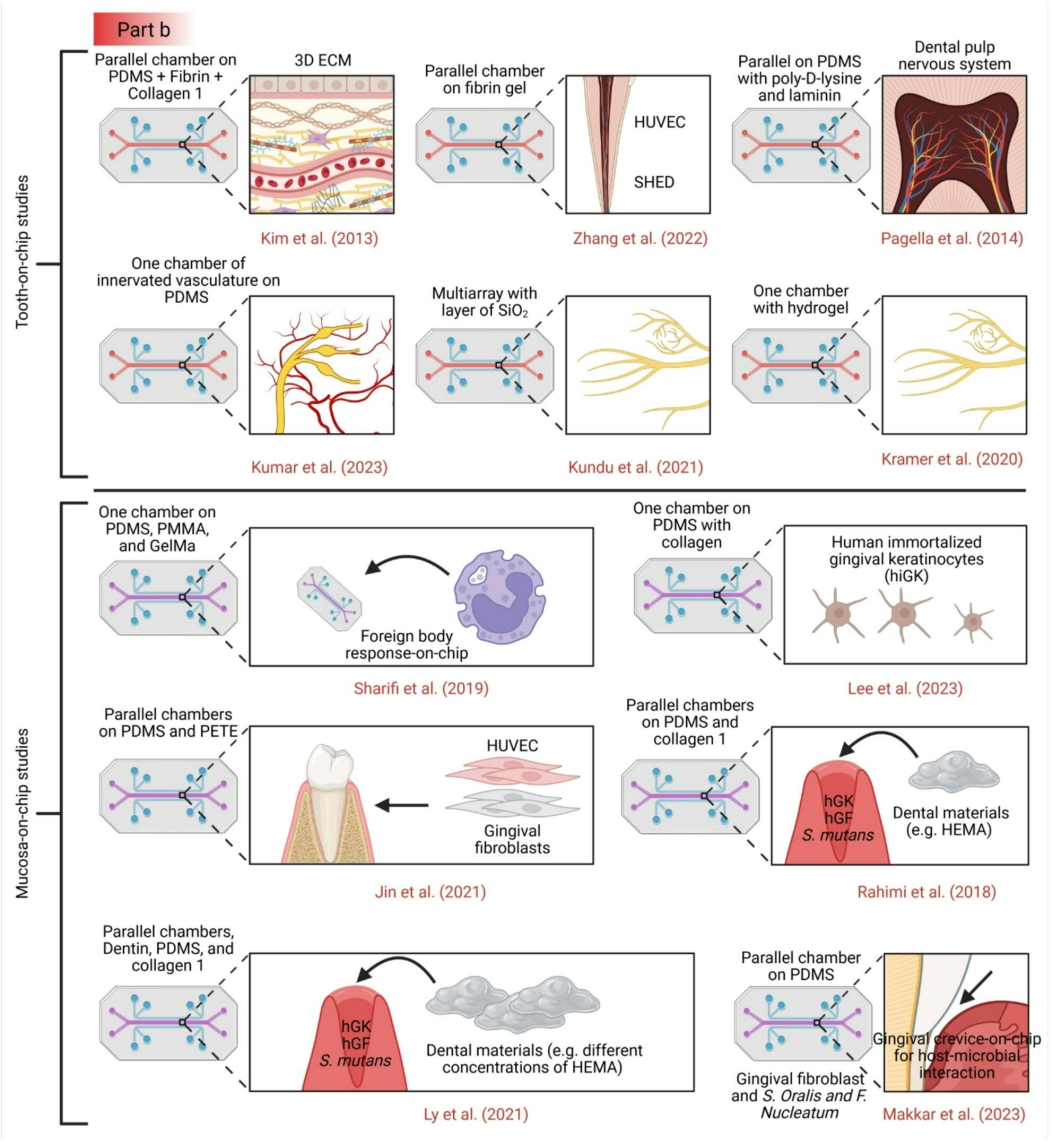

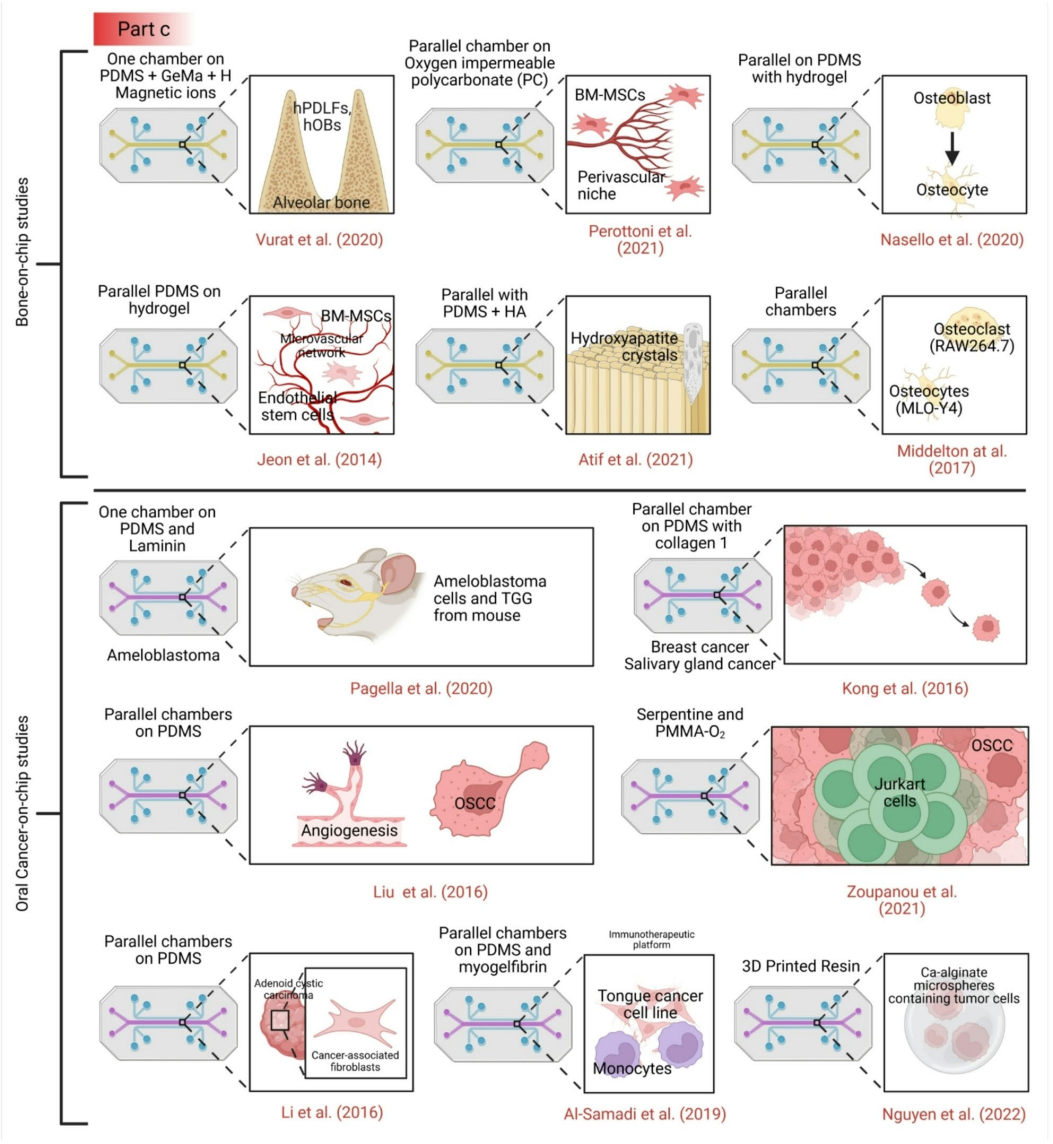

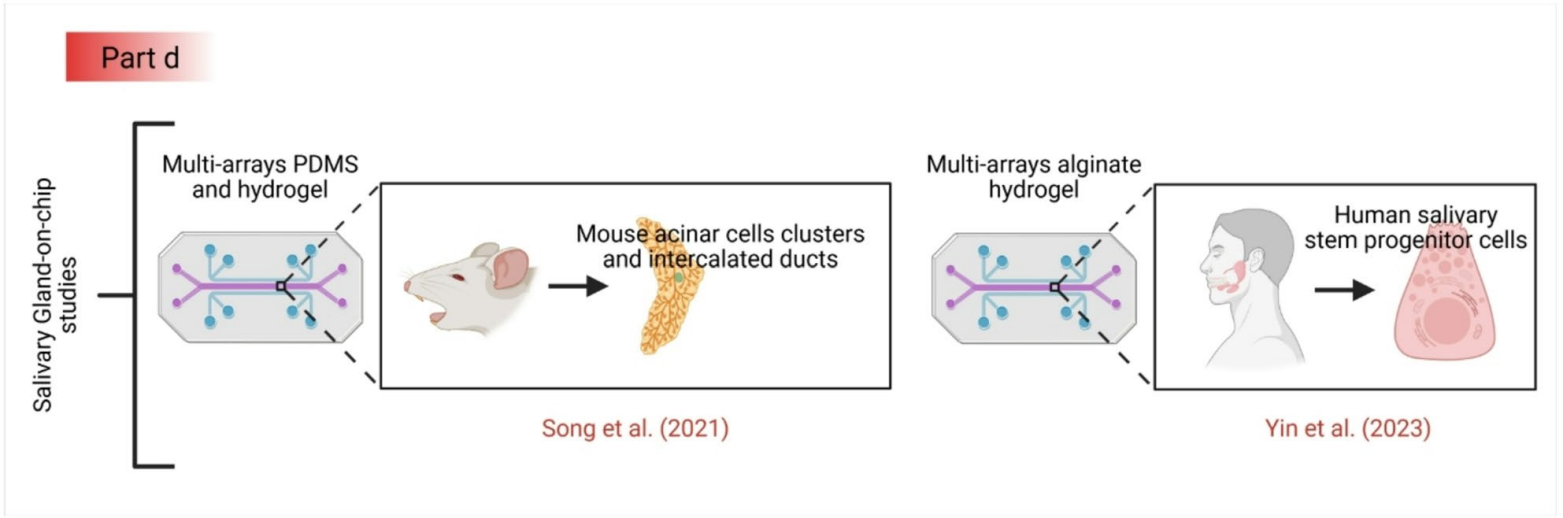
Current dental and craniofacial chip models. Part **a** and the upper panel of part b: Tooth-on-chip models [79], 85– [87, 89, 98], 117– [122]. Part **b** lower panel: Mucosa-on-chip models [91, 116], 123– [126]. Part **c**: bone-on-chip [127–132] and oral cancer on-chip models [133–139]. Part **d**: Salivary gland on chip models [140, 141]

Further limitations relate to the temporal and mechanical fidelity of current dental-on-chip systems [110–112]. Most models support only short-term cultures, restricting investigation of chronic processes such as long-term pulp inflammation, secondary dentinogenesis, peri-implant tissue remodeling, or peri-implantitis. Mechanical cues intrinsic to the oral environment, including mastication-induced strain and fluid shear within dentinal tubules, are rarely incorporated, and immune-microbiome interactions are still insufficiently represented. Implant-on-chip platforms that address host-material and pathogen-material interactions represent an important advance. Yet, they still lack systemic immune components and long-term perfusion necessary for chronic disease modeling and imitation. Addressing these limitations through the incorporation of immune cells, non-absorptive materials, perfusion systems, and standardized dentine substitutes will be essential to enhance translational relevance and readiness and to establish dental organ-on-chip platforms as reliable preclinical tools for regenerative dentistry and biomaterial testing (see Fig. 3) [113].

**Table 1:** Novel and state-of-the-art studies have investigated various dental tissues-on-chips

Reference	Targeted dental tissue	Device features	Cellular components	Key materials/ features
Ref [99]	Trigeminal innervated-teeth	Two chambers connected static microgrooved device	Mouse trigeminal ganglia cells, tooth germs, and postnatal mouse tooth	-
Ref [114]	OB process study in dentin hypersensitivity cases	Microchanneled microchip to mimic the OB processes and microchambers for OB culture	Odontoblasts	soft lithography-fabricated microchip
Ref [115]	PDL interaction with the implant surface	Static dual-insert microfluidic device with a single 400-µm-wide microchannel connecting a titanium (Ti/Ti-bHA) implant chamber and a calcium phosphate cement (CPC) chamber, incorporating a nanopatterned (400–800 nm) microgrooved PDMS substrate to guide PDL–like cell alignment under static culture conditions	PDL-like tissue derived from DPSCs	Dental implant, a calcium phosphate cement insert, a nanopatterned PDMS substrate, and
Ref [82]	Dental pulp	Multi-compartment static device composed of two main chambers (trigeminal ganglion and dental pulp compartments) connected by microgrooved microchannels	Co-culture of hDPSCs, OB-like cells, endothelial cells, and trigeminal neurones, allowing for the development of a vascular and neuronal network closely mimicking the structure and function of the tooth	-
Ref [81]	Dentin	Three-channel microfluidic chip with static 3D culture system	Short culture duration (≤ 7 days), no functional dentin-pulp interface or vascularization, limited material screening, and single cell type (no immune/endothelial components)	-
Ref [108]	Dental implant-on-a-cip for host-material pathogen interaction in peri-implant diseases	Spatially-separated co-culture with multiplexed microfluidic architecture	Keratinocytes, fibroblasts, gingival cells	dental resin, and biomaterials.
Ref [116]	Oral mucosa	Three microchannel networks, multi-layered tissue geometry, accessible layer-specific information, and higher sensitivity in detecting cellular responses to monomers like HEMA	Keratinocytes and fibroblasts	Collagen hydrogel embedding
Ref [106]	Pulp innervation (Model)	Three-channel microfluidic chip, micropillars, high-resolution confocal imaging, and quantitative angiogenesis analysis, like sprout length and branching, are among the advances of the current study The authors used HUVECs rather than pulp endothelial cells. Also, the absence of immune and neural cells, integrated dentin barriers, and the absorption of hydrophobic drugs by PDMS may present limitations of the study	DPSC, and HUVECs	-

### Gingiva-on-a-chip models

To model the mucosal barrier, researchers have developed a gingiva-on-a-chip platform. These typically involve culturing gingival keratinocytes and fibroblasts on porous membranes within microfluidic devices [142–144]. A notable example is the “*gingiva-on-a-chip*” developed by Gard et al., which successfully mimicked the stratified epithelial structure and demonstrated responses to inflammatory stimuli [144]. Other models have incorporated immune cells to study the host response to bacterial toxins [143]. However, these systems often operate in isolation, lacking the continuous challenge of a dynamic oral biofilm or the mechanical support of the underlying bone, limiting their physiological relevance to the complex periodontal environment [145, 146].

### Tooth-on-a-chip models

**Fig. 4:**
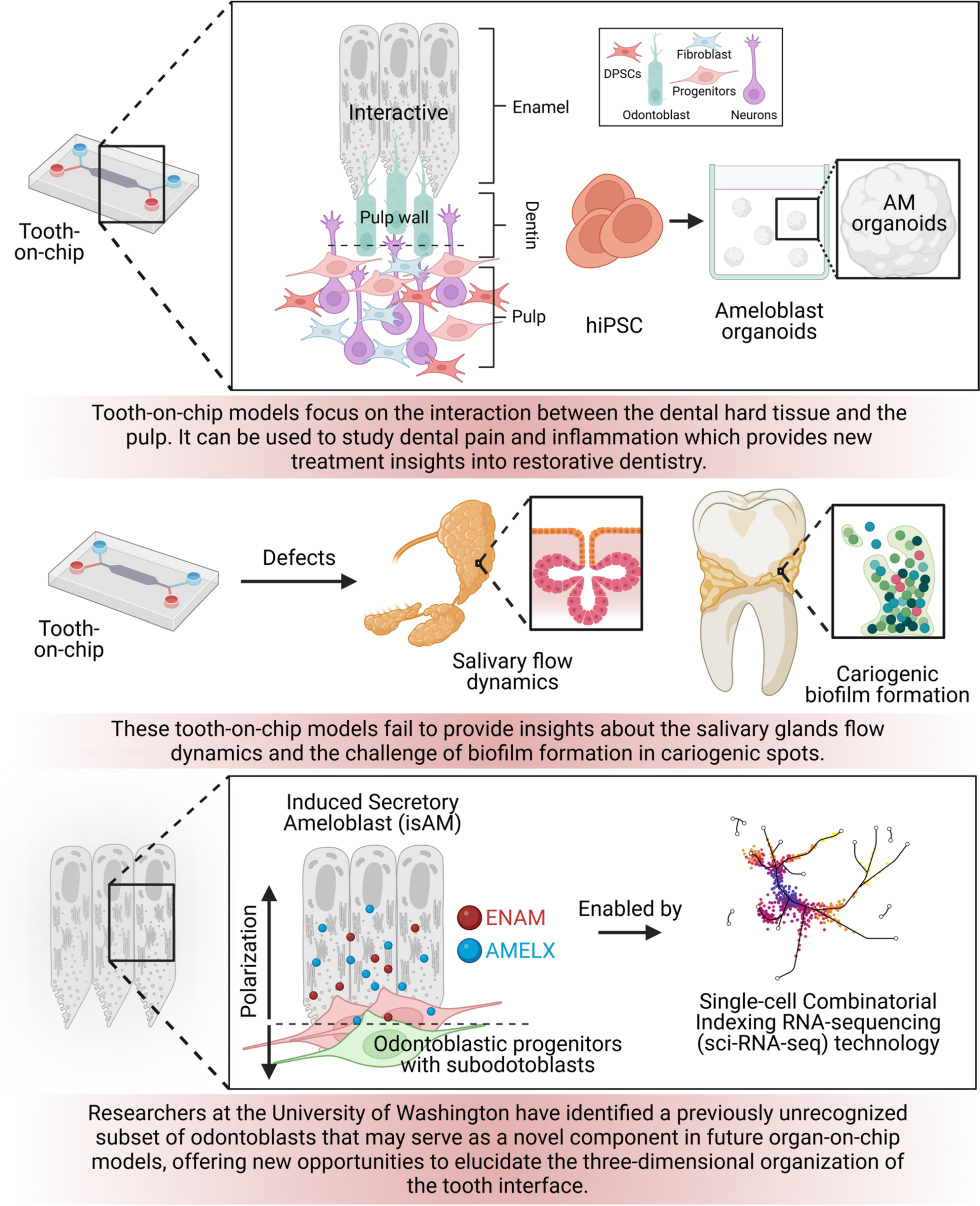
This figure illustrates the current capabilities, limitations, and recent and future directions of tooth-on-chip models. The top panel shows how these microfluidic devices simulate interactions between dental hard tissues (enamel and dentin) and the pulp, using diverse cell types, including stem cell-derived ameloblast organoids, to study dental pain and inflammation. The middle panel highlights current defects in these platforms, specifically their inability to accurately replicate salivary gland flow dynamics and the challenges they pose for cariogenic biofilm formation. The bottom panel demonstrates a recent advancement in which single-cell combinatorial indexing RNA-sequencing (sci-RNA-seq) was used to identify previously unrecognized subsets of odontoblastic progenitors and induced secretory ameloblasts; integrating these newly identified polarized cell populations into future models offers a promising pathway to better replicate the complex 3D organization of tooth structure

Tooth-on-a-chip models focus on the interaction between the dental hard tissues and the pulp (see Fig. 4) [82]. Devices integrating dentin slices have been used to study the permeation of dental materials and the response of pulp cells to varying cytotoxic agents. The “tooth-on-a-chip” by Cordiale et al. simulated the dentin-pulp interface to investigate the mechanism of dental pain and inflammation [82]. While this model innovatively includes vascularization and innervation, its reliance on extracted dentine introduces batch variability, and the lack of a dynamic biofilm compartment prevents its use in studying caries, a primary driver of pulpitis [59]. Interestingly, authors at the University of Washington have identified a novel subset of odontoblasts, called subodontoblasts, that may play another role in the development of microphysiological systems for various congenital diseases, such as amelogenesis imperfecta [110, 147–149].

### Oral mucosa-on-chip

The oral mucosa is a mucous membrane composed of stratified squamous epithelium, a basement membrane, and an underlying lamina propria. The epithelium may be keratinized or non-keratinized, depending on its oral location, while the basement membrane and lamina propria provide structural support and nourishment [116]. Oral epithelial cells function as a protective barrier, actively participate in inflammation, and directly interact with dental materials. Accordingly, in vitro mucosal cell models are widely used to study oral biology, disease, and responses to dental therapies, and should closely mimic native oral mucosa [145].

Li et al. have reported that the mucosa-chip is a promising platform that possesses a multi-layered tissue configuration of oral mucosa, mimics the physiological conditions of the oral mucosa interface, and allows for direct observation of mucosal cells’ responses to dental materials, such as HEMA. Compared to a traditional well-plate platform, the mucosa chip exhibits higher sensitivity in assessing cell layer-specific responses to the dental monomer HEMA in a dose-dependent manner within a physiologically relevant range [116]. Li et al. suggest that the mucosa-chip is a promising alternative to traditional testing platforms for evaluating the biocompatibility of dental materials [116]. Oral Mucosa-on-Chip models continue to face significant limitations. Many current systems replicate only the epithelium layer and lack essential components such as blood vessels, immune cells, bacteria, and dynamic salivary flow, all of which are crucial for accurately mimicking the natural oral environment. To enhance their utility for disease modeling and therapeutic evaluation, it is imperative to develop more integrated, physiologically relevant platforms [150].

### Periodontal ligament and periodontium-on-a-chip models

Periodontal ligament-on-a-chip platforms represent a significant advancement in periodontal research, as they faithfully recapitulate the complex biomechanical, cellular, and biochemical microenvironment of the native periodontal ligament (PDL). Svanberg et al. (2024) demonstrated that patient-derived periodontal ligament cells (PDLCs) actively regulate vascular morphogenesis by promoting the formation of stable, perfusable microvascular networks when co-cultured with endothelial cells in a 3D fibrin matrix under controlled perfusion conditions [97]. PDLCs exhibited pericyte-like behavior, physically aligning and wrapping around endothelial vessels, resulting in reduced vessel diameter, enhanced network maturation, and significantly improved endothelial barrier integrity compared with endothelial monocultures. Continuous flow further enhanced vascular perfusability and reproducibility, highlighting the critical role of shear stress in PDL microphysiology. Upon LPS stimulation, the chip recapitulated early inflammatory events characteristic of periodontitis, including upregulation of IL-6, IL-8, and IL-18, increased endothelial permeability, and elevated expression of adhesion molecules such as ICAM-1 and E-selectin, while maintaining overall extracellular matrix integrity. Additionally, besides the authors [151], Svanberg et al. (2024) have proposed the emergent need for the “Vasculature-on-chip” model as a complementary model for “*PDL-on-chip*.” [97].

**Fig. 5:**
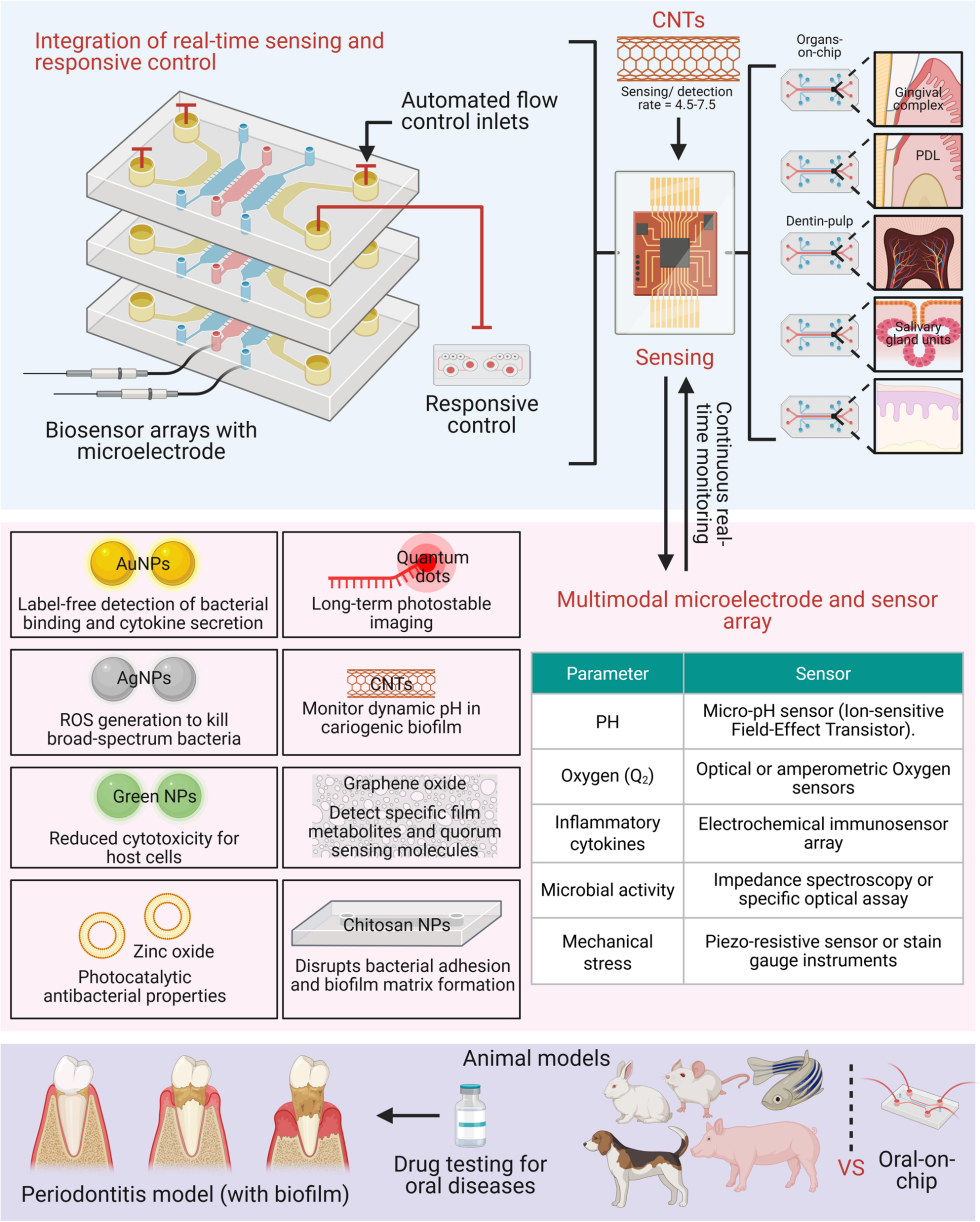
This figure presents a comprehensive overview of an integrated smart oral-on-chip platform equipped with real-time sensing and responsive control capabilities. The system utilizes multilayer biosensor arrays with microelectrodes and automated flow control to continuously monitor diverse microfluidic organ models, including the gingival complex, periodontal ligament, dentin-pulp, and salivary glands. Various functional nanomaterials, such as gold nanoparticles, quantum dots, carbon nanotubes, silver nanoparticles, and graphene oxide, are incorporated for specific biomedical applications ranging from long-term imaging and dynamic pH monitoring to broad-spectrum antibacterial action and metabolite detection. Furthermore, the platform features a multimodal microelectrode and sensor array designed to measure vital parameters like pH, oxygen levels, inflammatory cytokines, microbial activity, and mechanical stress using specialized sensors. This smart system provides a highly controlled environment for evaluating disease states like a periodontitis biofilm model and conducting drug testing, serving as a promising and targeted alternative to traditional animal models

Svanberg et al. (2024) model lacks key in vivo components, including immune cell populations, neural innervation, and resident oral microbiota, which limits its ability to recapitulate late-stage periodontal inflammation and host-pathogen dynamics fully (see Fig. 5). In addition, the simplified mechanical environment and absence of mineralized interfaces, like cementum and alveolar bone, constrain the study of load-dependent remodeling and long-term tissue degeneration [97].

### Salivary glands-on-a-chip (salivary gland mimics)

Salivary glands consist of three major glands, including the parotid gland (PG), submandibular gland (SMG), and sublingual gland (SLG), and numerous minor salivary glands [152, 153]. Additionally, a team of researchers in the Netherlands has reported the discovery of a new pair of salivary glands in the posterior nasopharynx called the Tubarial glands, with tissue like the palatal salivary glands [154]. The three major salivary glands are responsible for approxiamtely 90% of saliva production [155]. Acini, ducts, and myoepithelium are essential for producing and secreting saliva, which is mandatory for lubrication, digestion, immunity, and oral homeostasis [153, 156, 157]. In humans, serous acinar cells in the PGs secrete watery saliva, whereas the SLGs are predominantly composed of mucous acini that produce viscous, mucin-rich saliva; the SMGs contain both serous and mucous acini [158]. The three major salivary glands exhibit distinct cellular heterogeneity in saliva production but share common phenotypic features, including ducts composed of luminal and basal cells and acini surrounded by myoepithelium.

Salivary gland diseases encompass infections, such as COVID-19 [159], obstructions, autoimmune disorders, and tumors affecting saliva production [160, 161]. Common issues include sialadenitis, often bacterial, caused by *Staphylococcus*, due to blocked ducts or reduced flow, leading to painful swelling [162, 163]. Sialolithiasis involves calcium stones blocking the glands, leading to inflammation, while viral causes, such as mumps, target the parotid gland [164]. Extensive research papers on autoimmune conditions, such as Sjögren’s syndrome [165, 166]. It has been shown that self-immunity destroys the moisture glands, leading to many conditions like xerostomia [167–169].

**Fig. 6:**
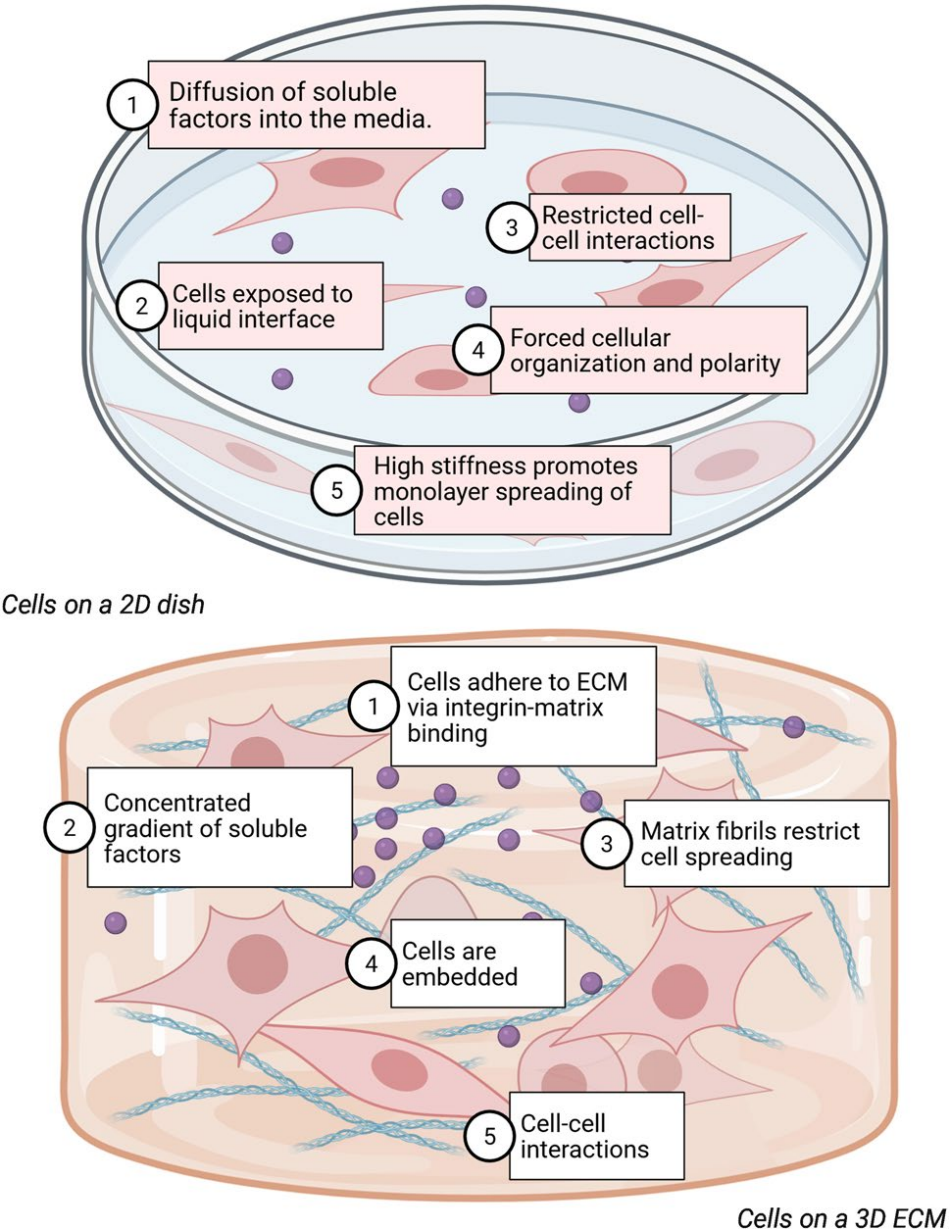
Comparison between 2D and 3D tissue cultures. All figures have been created from BioRender.com

Salivary gland research relies on experimental models, including animal systems, organoids, and patient-derived xenografts (PDX) [141, 170], but these have key limitations, driving a shift to advanced microphysiological systems such as salivary gland-on-a-chip. Organoids from murine/ human glands and PDX maintain gland-specific gene expression, histology, and functions like secretion, enabling long-term culture and scRNA-seq analysis [152, 171, 172]. Animal models suffer from species differences, such as rodent glands with more serous acini, prominent convoluted tubules absent in humans, varying radiosensitivity, ethical concerns, high costs for large animals such as minipigs or monkeys, and incomplete replication of human pathologies, such as inconsistent acinar loss or regeneration. 2D cultures lose cell polarity, phenotypes, 3D architecture, dynamic gradients, and vascular/ innervation elements, limiting microenvironment fidelity (see Fig. 6) [173–175].

Salivary gland-on-a-chip uses mild tissue dissociation and engineered matrices, such as collagen or Matrigel, in microwell arrays to culture heterogeneous salivary gland mimetics (SGm) from mouse or human tissues, preserving the architectural composition of cells, polarity, and functions, such as fluid secretion, for extended periods [141, 176–178]. This addresses gaps in animal and organoid models by mimicking vascular flow, innervation, and 3D dynamics, bypassing challenges in stem cell differentiation, and supporting high-throughput drug testing or disease modeling.

For instance, Piraino et al. conducted a recent study that tackles radiation-induced hyposalivation in head and neck cancer patients, a side effect that severely impacts eating, speaking, and oral health [141, 177]. As *Amifostine* is the sole FDA-approved drug for preventing radiation-induced hyposalivation, its severe side effects make it poorly tolerated, spurring the search for better alternatives. Hence, the SGm tissue chip platform, loaded with primary mouse submandibular gland cells in PEG hydrogels, was used to screen FDA-approved drugs for radioprotection [141, 177]. Other drugs reported to be radioprotective were tested, including *Tempol*, *Edaravone*, *N-acetylcysteine*, *Palifermin*, *Ex-Rad*, and *Rapamycin*. These tissue chip enables high-throughput, high-content screening of hundreds of drugs in a physiologically relevant 3D model that recapitulates native gland architecture, polarization, and radiation responses. It bypasses *Amifostine*’s limitations and accelerates translation to safer therapies for xerostomia, improving radiotherapy outcomes [141, 177].

## Limitations

**Fig. 7:**
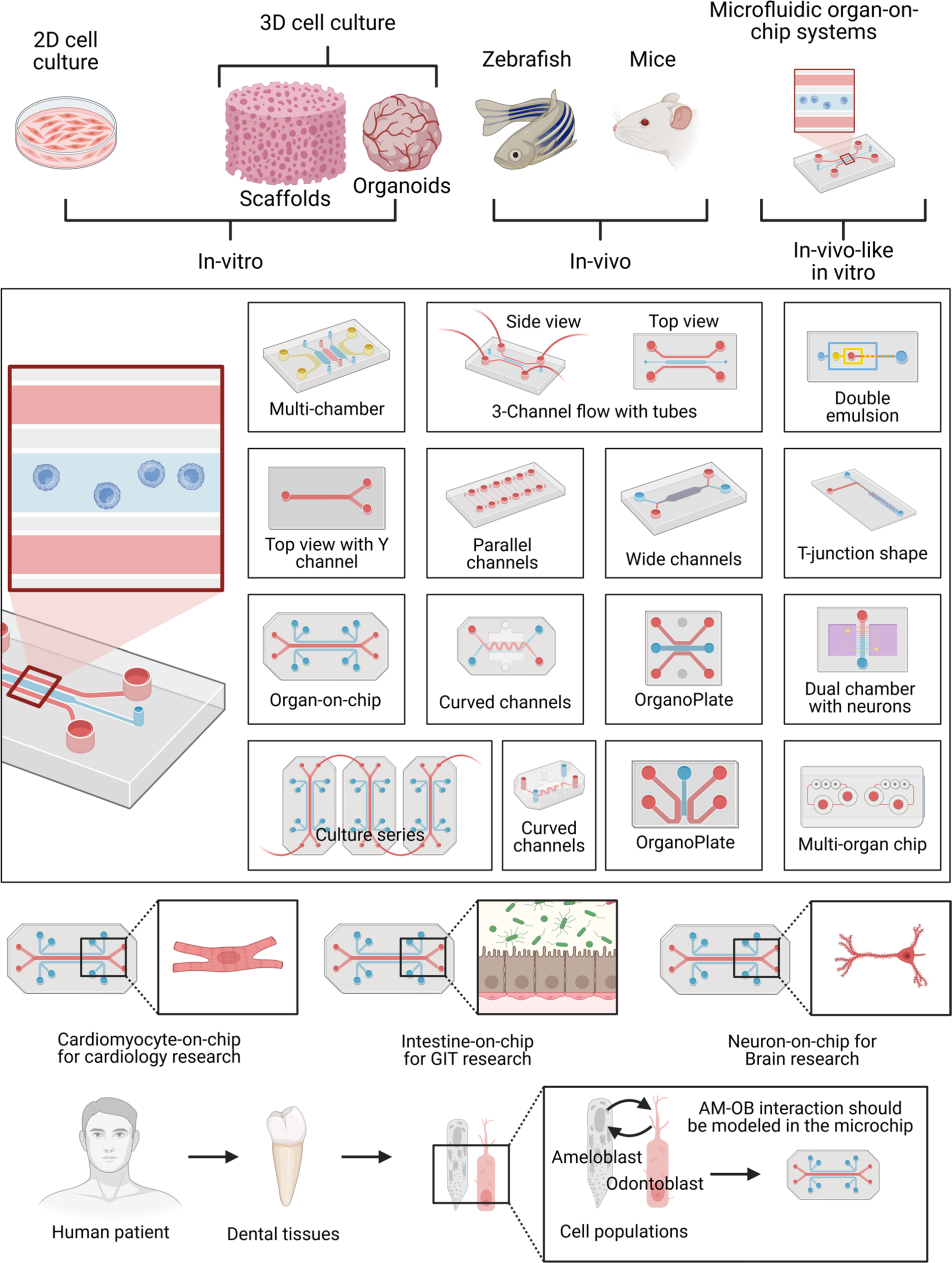
Experimental models and microfluidic design strategies supporting the transition toward organ-on-a-chip technologies in biomedical and dental research. The figure presents a comparative overview of conventional and advanced experimental platforms used to study human physiology and disease. Traditional in vitro approaches include 2D monolayer cell cultures and 3D culture systems, such as scaffold-based constructs and organoids, while in vivo models include zebrafish and murine systems. These platforms are contrasted with microfluidic organ-on-a-chip systems, which function as in vitro-like models that integrate key structural, biochemical, and dynamic features of living tissues within precisely controlled microscale environments. The central panel illustrates a range of commonly used microfluidic chip architectures, including multi-chamber devices, parallel and wide-channel systems, Y-shaped and T-junction geometries, curved channels, double-emulsion platforms, Organoplates, serial culture designs, dual-chamber neuronal models, and multi-organ chips, highlighting their versatility for modeling tissue interfaces, fluid flow, and cell-cell interactions. Representative applications of organ-on-a-chip technology include cardiology (cardiomyocyte-on-chip), gastrointestinal research (intestine-on-chip), and neuroscience (neuron-on-chip), demonstrating the broad translational potential of these systems. The lower panel emphasizes the extension of these concepts to dental research, depicting the progression from human patients to dental tissues and relevant cell populations, and the incorporation of odontogenic cell interactions, such as ameloblast-odontoblast crosstalk, within microfluidic platforms. All figures have been created using BioRender.com

Despite the increased number of human and dental tissues integrated into chips and their advances (see Fig. 7), current organ- and oral-on-chip platforms suffer from three fundamental limitations. First, existing chip models focus on either biofilm or host tissue, but not on their dynamic crosstalk, failing to capture the complete disease pathology. Additionally, most platforms rely on traditional endpoint assays (e.g., staining, *ELISA*) and lack real-time monitoring of critical parameters [179], such as pH, bacterial metabolites, or cytokines in other organs [180–183]. For instance, human bronchial epithelial cells cultured within a microfluidic platform were exposed to lipopolysaccharide (LPS), and their cytokine release was monitored in real time [184]. The system also captured analyte transport across the tissue compartment following barrier disruption. This study represents the first integration of photonic sensing with a human tissue-chip device, enabling advanced applications in drug discovery and disease modeling [184]. Also, the heavy reliance on PDMS and non-biodegradable polymers contradicts the growing mandate for sustainability in precision oral healthcare technologies [185]. Due to such limitations, the convergence of biologically compatible nanomaterials with oral microfluidic devices has emerged, driven by nanosensors and their applications.

## The convergence: nanobiomaterials in oral microfluidics

### Nanosensors for real-time monitoring

The integration of nanobiomaterials into microfluidic systems represents a pivotal step toward next-generation OoC platforms [179]. Nanomaterials offer unique optical, electrical, and mechanical properties that can enhance sensing capabilities [186, 187], enable precise actuation [188], repurpose different cell populations [46], and provide biomimetic scaffolds [71]. Real-time monitoring of the microenvironment is crucial for capturing dynamic physiological events. Carbon nanotube (CNT)-based sensors have been integrated into microfluidic channels to provide high-sensitivity pH monitoring (detection range: 4.5–7.5) with millisecond response times, which is essential for tracking acidification in cariogenic biofilms [189]. Gold nanoparticles (AuNPs) that utilize localized surface plasmon resonance (LSPR) enable label-free detection of bacterial binding and cytokine secretion [190]. Furthermore, graphene oxide electrochemical sensors have demonstrated the ability to detect specific biofilm metabolites, such as quorum-sensing molecules [191]. Quantum dot fluorescent probes allow for long-term, photostable imaging of cellular components and cytokine gradients within the chip [192].

Recent advances in stimuli-responsive and biomimetic nanobiomaterials are broadening the scope of nanotechnology’s oral microphysiological applications. In terms of sensing materials, this is superior to CNTs and AuNPs. Microfluidic oral models use stimuli-responsive hydrogels and polymeric nanoparticles for controlled antibacterial release and targeted therapy. This improves the simulation of the cariogenic and periodontal microenvironment. These advances also enable the integration of dental biomimetic materials [193].

Polydopamine and hydroxyapatite-based nanocomposites are widely used in dental implantology and restorative studies because they enhance osseointegration, antibacterial properties, and tissue compatibility. When used with oral microfluidic devices, these materials enable realistic interactions between implants and tissues, as well as the dynamics of biofilms. These advances show that intelligent biomaterials and microphysiological technology are converging [194].

### Nanomaterials for biofilm control

Nanomaterials are not just passive sensors but active modulators of the biological environment. Silver nanoparticles (AgNPs) are widely used for their broad-spectrum antimicrobial activity, mediated by ROS generation and membrane disruption [195]. Zinc oxide nanoparticles (ZnO-NPs) exhibit photocatalytic antibacterial properties, which are useful for self-cleaning chip surfaces [196, 197]. Chitosan nanoparticles, with their cationic charge, can disrupt bacterial adhesion and biofilm matrix formation [198, 199]. Additionally, green-synthesized nanoparticles derived from plant extracts offer a sustainable route to producing antimicrobial agents with reduced cytotoxicity for host cells [200, 201].

### Nanocarriers for precision drug delivery

To simulate therapeutic interventions, nanocarriers enable precise spatiotemporal drug delivery within the chip. Liposomal nanocarriers have been employed to deliver antimicrobial peptides directly to biofilm niches [202]. Poly(lactic-co-glycolic acid) (PLGA) nanoparticles provide sustained release profiles, allowing researchers to model chronic drug exposure [203].

Priti P. et al. demonstrated that nanoparticle-based systems can enhance localized drug absorption and antimicrobial activity in dental tissues, highlighting the potential of functional nanobiomaterials for targeted therapeutic delivery in oral applications [204]. Stimuli-responsive nanocarriers, triggered by pH drops or specific bacterial enzymes, allow for “smart” drug release only in pathogenic microenvironments [205–209]. Biomimetic nanoparticles, along with biomimetic microenvironments [210] used in regenerative endodontics [211], pulp regeneration [212], craniofacial applications [213], periodontal tissue engineering [214], and periodontium regeneration [215], and oral medicine [216], such as cell membrane-coated nanocarriers, mimic host cells and tissues to serve as entities for bacterial toxins.

### Sustainable nanobiomaterials

The reliability and predictive capabilities of organ-on-chip (OoC) systems are significantly influenced by material selection. The ability of the materials used in devices to maintain stable biochemical conditions and provide appropriate drug concentrations within the microfluidic environment is essential for precision medicine and drug discovery. The significance of material fidelity has increased due to the substantial impact that material-related artifacts can have on pharmacological assays and biological reactions. Due to its ability to adsorb small hydrophobic compounds, including many pharmaceuticals, PDMS limits the performance of microfluidic systems. The hydrophobic polymer network allows these molecules to diffuse into the substance, reducing the culture medium’s drug concentration. The intended and actual drug dosages cells encounter may differ, affecting dose–response relationships and the reproducibility of pharmacological tests, especially in high-throughput drug screening and precision medicine. Several sustainable bio- and nanomaterials have been utilized in conjunction with different organs-on-chips [40, 217–219] (see Table 2). For instance, cellulose nanocrystals (CNC) are emerging as robust, biodegradable alternatives to PDMS, offering high optical transparency and mechanical strength [220]. Alginate-based microfluidic devices provide a fully biodegradable platform suitable for short-term culture [221]. Silk fibroin nanofibers can be processed into biocompatible scaffolds and membranes for tissue interfaces [222]. Bacterial cellulose membranes produced via green synthesis exhibit excellent mechanical properties and biocompatibility, making them suitable for soft tissue modeling [223].

Recent developments in dental materials research have shifted the focus away from passive restorative materials toward bioactive and intelligent biomaterials capable of interacting with the biological environment [69]. Polymers that respond to stimuli, antimicrobial nanocomposites, and bioactive implant coatings are among the new materials developed [224]. These coatings are intended to promote osteointegration, control inflammation, and prevent bacterial biofilm formation. Restorative and implant materials are increasingly being developed to perform therapeutic and regenerative functions rather than serving solely as structural alternatives. This signifies a notable shift in modern dentistry, marked by the utilization of these materials [225].

**Table 2:** Various organ-on-chip devices have been developed in recent years

Bio- and nanomaterial	Organ-on-chip	References
PDMS	Lung- and airway-on-chip	[226]
PDMS / PC membrane	Proximal renal tubes-/ multitubular kidneys-on-chips	[227]
PDMS	Liver-on-chip	[228]
Gelatin Methacryloyl (GelMA) Hydrogel	Heart-on-chip	[229]
Polycarbonae (PC) and PDMS		[230]
Microporous PDMS	BBB-on-chip	[231]
Acetazolamide (AZA), Edaravone (EDA), and Fasudil (FAS)	Ischemia-reperfusion injury in stroke-on-chip	[232]
Polyethylene Glycol Hydrogel	BBB	[233]
Polymeric nanoparticles	BBB-on-chip for neurological diseases	[234]
Aptamer nanoconstructs for nanosuttle discovery	BBB-on-chip for BBB cross mechanics	[235]
Biosensores	BBB-on-chip model	[236]
Tetrahedral DNA Frameworks for LPS-mediated inflammation visualization	µF-hBBB Chip	[237]
Gold nanorods	BBB‑on‑a‑chip with integrated micro‑TEER for AD	[28]
cerium-peptide composite microsphere	Two-syringe microfluidic chip	[238]
OSCC diagnosis aided by antibody-grafting microbeads	OSCC-fuild-on-chip	[239]

### The integration strategies of nanobiomaterials in microfluidic devices for personalized medicine

Organ-on-chip technology holds great promise as a complementary technology to animal experimentation and as an effective tool for implementing the 3Rs (reduction, refinement, and replacement). For instance, OoC systems can enable a superior a priori design of experiments and therefore reduce the number of animal trials with statistically insignificant results [240–244]. In addition, compared with animal models, OoC systems are advantageous for providing predictive models for human-specific physiological and pathophysiological studies. Furthermore, including patient-derived cells in OoC offers significant potential for drug development for rare diseases, clinical experiments, and even the transition from one-size-fits-all therapies to personalized medicine approaches [240–244]. The integration of novel, biocompatible nanomaterials into microfluidic systems offers an opportunity to develop disease-specific models (see Table 3).

**Table 3:** Current nanobiomaterials are integrated into the microfluidic devices

Nanomaterial	Advantages	Limitations	Reference
Chitosan nanoparticles	Sustained release, enhanced adhesion, osteoinduction	Swelling, channel blockage	[245]
PLGA nanoparticles	Controlled delivery, tunable degradation	Acidic byproducts	[246]
Gelatin nanoparticles	Growth-factor delivery	Thermal instability	[247]
Collagen nanofibrils	Native ECM mimicry	Low stiffness, variability	[248]
Alginate nanogels	Encapsulation, biocompatibility	No adhesion motifs	[249]
PEGDA nanoparticles	Anti-fouling, tunable stiffness	Non-degradable	[250]
Gold nanoparticles	Biosensing, osteogenic signaling	Aggregation risk	[251]
Silver nanoparticles	Antimicrobial	Cytotoxicity	[252]
Titanium dioxide nanoparticles	Improved adhesion	ROS generation	[253]
Iron oxide nanoparticles	Magnetic manipulation	Accumulation risk	[254]
Magnesium nanoparticles	Osteoinductive ions	Gas evolution	[255]
Hydroxyapatite nanoparticles	Bone mimicry	Agglomeration	[256]
Bioactive glass nanoparticles	High bioactivity	Alkalinity	[257]
Carbon nanotubes	Electrical conductivity	Aggregation, toxicity	[258]
Carbon quantum dots	Fluorescent tracking	Lower brightness	[259]
Nanodiamonds	Stable drug carriers	Channel abrasion	[260]
Nanoclays (Laponite)	Rheological control	Dose-dependent cytotoxicity	[261]
Graphene quantum dots	Stable photoluminescence	Synthesis complexity	[262]
Polyurethane nanofibers	Elasticity	Potential leachables	[263]
PLA-based nanocomposites	Biodegradability	Thermal sensitivity	[264]
Cellulose-HA composites	High water retention	Limited infiltration	[265]

Recent literature (2020 and 2026) has investigated numerous nanomaterials in many research papers, as discussed in the perspective table

## Recent studies integrating nanotechnology in microfluidic design and preparation

Recent advances collectively demonstrate that the convergence of microfluidics, nanomaterials, and biomolecular engineering is laying the foundation for increasingly sophisticated organ-on-chip platforms directly translatable to dental research. Micro- and nanofluidic architectures now enable precise spatial control, dynamic flow, and real-time analysis, as shown by protocell communication systems based on DNA nanotechnology that rely on microfluidic trapping to arrange collective signaling behaviors [266], nanofluidic devices that continuously assess biologic drug quality under flow [267], and nanoparticle-enabled microfluidic chips that phenotype rare cancer cells with single-cell resolution [266, 268]. These approaches conceptually support dental organ-on-chip models, such as pulp-, gingiva-, or oral cancer-on-chip, where controlled molecular transport, intercellular communication, and phenotypic heterogeneity are critical features. Complementary nano-enabled microfluidic electrophysiology platforms further demonstrate how nanoscale electrodes integrated into chips can record functional cellular activity in intact biological systems, providing a blueprint for innervated dental tissue models that couple neural signaling with engineered biomaterial environments [269]. Hence, their applications could be in scaffold variations, iPSC-derived sources, and targeted enamel regeneration.

As scaffold variations and designs, nanomaterials, and nano-optical systems expand the analytical and therapeutic capabilities of organ-on-chip technologies, these technologies are highly relevant to dentistry. Metasurface optofluidic platforms and nanophotonic biosensors integrate nanostructured materials directly within microfluidic channels to enable dynamic optical sensing, label-free detection, and real-time monitoring of biochemical processes, offering clear opportunities for saliva-on-chip diagnostics, mineralization tracking, and biofilm analysis. Whereas targeted nanomedicine strategies exploit nanoparticle surface chemistry to selectively bind activated immune cells, further illustrating how nanobio interfaces can be evaluated under physiologically relevant flow, a concept directly applicable to periodontitis- and inflammation-on-chip models [269].

The nanopore-based single-molecule sensing platforms highlight how nanoscale confinement and microfluidic integration can achieve ultrasensitive detection of proteins, nucleic acids, and metabolites [270], reinforcing a unifying theme across these studies, including organ-on-chip systems enriched with nanomaterials that are evolving into multifunctional dental research platforms capable of modeling physiology, pathology, diagnostics, and therapeutic response within a single, controllable microscale device.

Recent studies further emphasize how nanoscale control of matter, transport, and biochemical reactions within microfluidic environments is transforming OoC platforms into precision experimental systems. Nanofluidic electrokinetic nanovalves enable the isolation, confinement, and manipulation of single nano-objects, including viruses, vesicles, and biomolecules, at physiological ionic strengths, offering unprecedented control over molecular transport directly relevant to dental pulp-, saliva-, and pathogen-on-chip models [271]. In parallel, plasmonic nanostructures integrated into autonomous microfluidic cartridges dramatically accelerate nucleic acid amplification and enable rapid, colorimetric detection of salivary pathogens, establishing a clear blueprint for saliva-on-chip diagnostics and chairside oral infection screening [272]. Complementing these advances, micro- and nanoscale device platforms developed for probing epigenetic and chromatin dynamics demonstrate how confined microenvironments and nanoscale interfaces can reveal gene-regulatory states from small cell populations, a concept directly applicable to studying epigenetic dysregulation in oral cancer and periodontal disease-on-chip systems [273].

At the same time, these works highlight how nanomaterials enhance both biological realism and translational relevance of chip-based models. Microfluidic technologies designed to synthesize, screen, and validate nanoparticles under physiologically relevant conditions address a critical bottleneck in nanomedicine translation and provide a framework for evaluating nano-enabled dental therapeutic directly on organ-on-chip platforms [274]. DNA-based nanoarchitectures, including bio-hybrid rotary nanoengines and DNA origami gene logic chips, demonstrate programmable biochemical computation and energy transduction at the nanoscale, suggesting future smart dental chips capable of localized sensing, gene regulation, or responsive biomaterial behavior. Finally, critical perspectives on cancer model validation underscore the need for accurate, benchmarked in vitro systems, positioning nano-enabled organ-on-chip models as essential intermediates between conventional cell culture and clinical dentistry, particularly for oral cancer drug testing and personalized therapeutic screening [275].

## Conclusion and future directions

In this review, we have outlined the critical need for advanced in vitro models that accurately reflect the complex physiology of the oral cavity. While numerous platforms have successfully recapitulated dental tissue microenvironments, such as the dentin-pulp interface and oral mucosa interactions, several limitations remain. Challenges related to material variability, device scalability, and the incorporation of tissue-specific phenotypes must be addressed to improve the physiological relevance of these models. Future research will integrate microfluidics, nanobiomaterials, and sustainable engineering to develop multi-layered intelligent organ-on-chips. This platform’s precise regulation of the mechanical, chemical, and biological milieu could transform understanding of oral diseases and expedite the development of targeted, effective medicines, heralding a new era of precision oral healthcare.

Subsequent investigations should concentrate on oral-on-chip systems capable of simulating the dynamic interactions between oral fluids and various prosthodontic apparatus, including restorations, crowns, and orthodontic devices. These devices would provide controlled investigations into the effects of mechanical loading, fluid shear, and oral microbiota dynamics on biofilm proliferation, surface degradation, and the long-term performance of materials at prosthetic interfaces. Native oral tissues, artificial dental devices, and therapeutically relevant restorative materials should be integrated into cohesive oral-on-chip platforms. A strong framework for systematically examining material-tissue–microbiome interactions under patient-specific oral settings would allow precision dentistry and better predictive preclinical dental biomaterial assessment with these next-generation models.

## Data Availability

No datasets were generated or analysed during the current study.
